# Percutaneous transluminal angioplasty vs. stenting for hepatic artery stenosis after liver transplantation in adults: a systematic review and meta-analysis

**DOI:** 10.1186/s42155-025-00581-8

**Published:** 2025-10-07

**Authors:** Mahmoud Shaaban Abdelgalil, Ahmad Abdelrazek, Adam Hraybi, Marwa Hassanien, Abid Wazir, Akram Elegili, Abdelrahman Abdelrazek, Hammad Tanoli, Sara Metwally

**Affiliations:** 1 Faculty of Medicine, Ain Shams University, Cairo, Egypt; 2 Hartford HealthCare, St. Vincent’s Medical Center, Bridgeport, CT USA; 3 University of Michigan-Dearborn, Dearborn, MI USA; 4 Faculty of Medicine, Assiut University, Assiut, Egypt; 5 University of Michigan Dearborn, Dearborn, MI USA; 6 Faculty of Medicine, Misr University for Science and Technology, Giza, Egypt; 7 Faculty of Medicine, October 6 University, Giza, Egypt; 8 Wayne State University, Detroit, MI USA; 9 Corewell Health Grosse Pointe Hospital, Grosse Pointe, MI USA

**Keywords:** Hepatic artery stenosis, Liver transplantation, Percutaneous transluminal angioplasty, Stent placement, Endovascular treatment

## Abstract

**Purpose:**

Hepatic artery stenosis (HAS) is a serious complication of liver transplantation (LT), with no established guidelines for choosing between percutaneous transluminal Angioplasty (PTA) and stent. This study compared their outcomes to inform clinical practice.

**Materials and methods:**

We searched PubMed, SCOPUS, Cochrane library, and Web of Science for studies comparing PTA and stenting in HAS patients after LT. The primary outcome was primary patency rate. Secondary outcomes included technical success, major complications, Hepatic artery thrombosis (HAT) incidence, reintervention rates, and time to recurrent HAS. We also conducted a subgroup analysis based on major complication types, specifically artery dissection and rupture.

**Results:**

Nine observational studies, including 325 patients with HAS after LT, were analyzed, with 140 treated with PTA alone and 197 with stents. No significant differences were found between stenting and PTA in primary patency rates at 6 months, 12 months, and the end of follow-up, as well as in technical success, major complications, artery dissection, artery rupture, and HAT incidence. However, stenting was significantly associated with a lower reintervention rate (RR = 0.57, 95% CI [0.36, 0.89], *P* = 0.01) and a longer time to recurrent HAS compared to PTA (MD = 36.42, 95% CI [14.14, 58.70], *P* = 0.001).

**Conclusion:**

Both PTA and stenting show similar primary patency and safety for HAS after LT. However, stenting offers lower reintervention rates and longer recurrence-free intervals, suggesting better long-term outcomes. Treatment selection should be individualized, considering anatomical factors, stenosis morphology, and operator expertise.

**Supplementary Information:**

The online version contains supplementary material available at 10.1186/s42155-025-00581-8.

## Introduction

Hepatic artery stenosis (HAS) is a well-recognized complication following liver transplantation (LT), with an incidence ranging from 3.5% to 11% [1–4]. If left untreated, HAS can progress to hepatic artery thrombosis (HAT) [5, 6] in approximately 65% of cases within six months [7, 8], potentially leading to ischemic cholangitis, graft failure, or even patient death [9]. HAS typically develops at the arterial anastomosis and has been associated with various factors, including allograft rejection, surgical technique, donor-recipient hepatic artery caliber mismatch, and microvascular injury related to cold ischemia time [10]. While HAS can occur at any point after LT, it is often asymptomatic until HAT develops, although it may sometimes present with biliary complications or graft dysfunction [11].

Traditional treatments for HAS and HAT have included open surgical arterial revascularization or retransplantation [12]. However, with advancements in minimally invasive techniques, endovascular therapy has become the first-line treatment. Both percutaneous transluminal angioplasty (PTA) [8, 13, 14] and stenting [15–17] have demonstrated favorable outcomes in terms of arterial patency, graft survival, and recipient survival, except in cases of early postoperative stenosis, which often requires surgical intervention [10]. Despite their widespread use, no established guidelines exist to determine the optimal approach between PTA and stenting for primary HAS treatment. While some studies, including a meta-analysis by Rostambeigi et al. [13, 18], have reported comparable outcomes for both techniques, however, others suggest that HAS recurrence, particularly after PTA, often necessitates repeated interventions [8, 12].

Although the meta-analysis by Rostambeigi et al. provided valuable insights into HAS management after LT, it had notable limitations [18]. It primarily included single-arm studies, limiting direct comparisons between PTA and stenting [18]. Additionally, it included studies up to 2011 [18], and more recent studies have since emerged [11, 12]. These newer studies may provide updated insights into the comparative efficacy and safety of PTA and stenting.

To address this gap, our meta-analysis aims to provide an updated and comprehensive comparison of PTA and stenting for the treatment of HAS after LT. By analyzing key parameters such as primary patency, reintervention rates, and major complications, we aim to offer clearer guidance for clinical decision-making and improve the understanding of the optimal endovascular approach for HAS management.

## Methods

This meta-analysis study was conducted in accordance with the Preferred Reporting Items for Systematic Reviews and Meta- Analyses (PRISMA) guidelines [19] and Cochrane Handbook [20]. The study protocol was submitted and registered in the PROSPERO database CRD420251016905.

### Literature search

A comprehensive search was conducted on January 28, 2025, using PubMed, Cochrane, Scopus, Web of Science, and Embase databases. The search strategy incorporated the following keywords: “Liver Transplantation,” “Post-Transplantation Complication,” “Stent,” “Angioplasty,” “Percutaneous Transluminal Angioplasty,” “Hepatic Artery Stenosis,” and “Hepatic Artery Narrowing.” A detailed description of the search strategy is available in the supplementary file.

### Eligibility criteria and study selection

We included randomized controlled trials and observational studies comparing PTA alone versus stenting for HAS after LT in adult patients. Studies on pediatric populations, case reports, cross-sectional studies, editorials, reviews, and studies not reporting at least one of our outcomes were excluded. Only English-language publications were considered.

To ensure a comprehensive and systematic selection process in title and abstract screening, we used Rayyan.ai, an AI-powered tool that facilitated study management, blind screening, AI-assisted tagging, and duplicate detection [21]. Two independent authors screened titles and abstracts for eligibility using Rayyan.ai. Studies meeting the criteria underwent full-text assessment, and if the full text was unavailable, we contacted the corresponding authors via email. Any discrepancies were resolved through consensus or consultation with a third author. Additionally, we manually reviewed reference lists and conducted backward citation analysis to identify any relevant studies.

### Data extraction and study outcomes

Two independent reviewers extracted data using a standardized Excel sheet. Collected data included study characteristics (e.g., author name, year, country, and study design), participant demographics (e.g., sample size, age, and male), and relevant clinical outcomes. Our primary outcome was the primary patency rate at 6 months, 12 months, and the end of follow-up. Secondary outcomes included technical success rate, major complication rates, incidence of HAT, incidence of reintervention, and time to recurrent HAS following endovascular treatment.

Primary patency rate was defined as the duration of vessel patency without the need for reintervention, while technical success rate referred to the successful completion of the endovascular procedure with restored hepatic artery flow. Major complications included arterial rupture and hepatic artery dissection. The incidence of HAT was defined as complete arterial occlusion following treatment, whereas the need for reintervention referred to the recurrence of significant stenosis requiring additional endovascular procedures, such as restenting or repeat percutaneous angioplasty. Finally, time to recurrent HAS was measured as the interval between the initial treatment and the recurrence of hepatic artery stenosis requiring intervention.

### Quality assessment and risk of bias

Two independent reviewers assessed the quality and risk of bias in cohort studies using the Newcastle–Ottawa Scale (NOS), which evaluates three key domains: selection (4 points), comparability (2 points), and outcome assessment (3 points), with a maximum score of 9. Studies scoring ≥7 was considered high quality [22]. Any discrepancies were resolved through consensus or consultation with a third reviewer.

### Data synthesis and statistical analysis

We conducted statistical analyses using Review Manager (RevMan) 5.4.1. For continuous variables, mean difference (MD) was calculated, while risk ratios (RR) were used for dichotomous outcomes. Statistical significance was set at *P* < 0.05. Initially, a fixed-effects model was applied; however, when heterogeneity was detected, we adopted a random-effects model and performed a leave-one-out sensitivity analysis. Heterogeneity was evaluated using the I^2^ statistic (> 50% indicating significant heterogeneity) and *P*-values (< 0.1) [23]. We also conducted a subgroup analysis based on major complication types, specifically artery dissection and rupture. Due to the inclusion of less than 10 studies, we were unable to assess publication bias.

## Results

### Literature search results

**Fig. 1:**
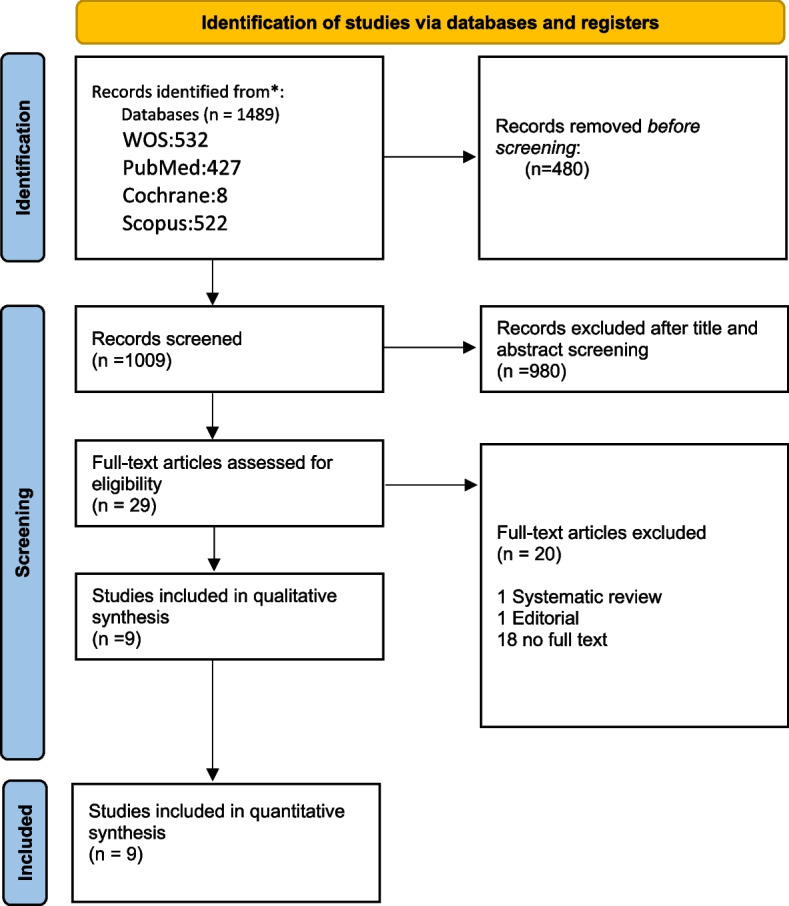
PRISMA flow diagram for included studies

Our extensive search initially identified 1489 records. After removing duplicates, 1009 records remained for title and abstract screening. Of these, 29 articles were deemed potentially eligible for full-text review. Ultimately, 9 observational studies met the inclusion criteria and were included in our systematic review and meta-analysis. The PRISMA flow diagram is presented in Fig. 1.

### Characteristics of included studies

Our analysis included nine studies with a total of 5386 liver transplant recipients, among whom 309 (5.73%) developed hepatic artery stenosis (HAS). However, Sabri et al. [24] was excluded from the HAS incidence calculation due to the lack of reported liver transplant numbers in their study (Table 1). As a result, the total number of patients with HAS across the included studies was 325, with 140 undergoing PTA alone and 197 undergoing stents.

**Table 1:** Shows a summary of the included studies

Study ID	Country	Type of the study	Sample size of each group	Age (years) Mean ± SD	Gender (Male) n	Inclusion criteria	Study duration	Number of liver transplant	The incidence of HAS	The mean time from transplantation to vascular intervention	Conclusion
**Khati et al. 2020** [25]	France	Retrospective	17 patients, (PTA *n* = 5, stents *n* = 5, PTA + stent *n* = 7)	57	14	Liver transplant recipients with significant HAS diagnosed via ultrasound	2013–2020	413	4% (17/413)	178.5 days	This study suggests that PBA, stent, or both procedures show the same primary patency at 12 months
**Magand et al. 2019** [11]	France	Retrospective	42 patients,51 EVT: PTA alone n = 34 and stents *n* = 16)	PTA = 55.4 ± 10.5 Stent = 54.9 ± 14.0	PTA = 26 Stent = 12	Liver transplant recipients diagnosed with HAS via ultrasonography	1999–2017	1331	3.9% (52/1331)	387 days	Both PTA and stenting were effective; however, there was a strong trend toward higher HAS recurrence after PTA than after stenting, suggesting that primary stenting should be preferred for long-term patency
** Goldsmith et al. 2017 ** [26]	USA	Retrospective	99 patients: PTA alone (*n* = 34) or with stent (*n* = 65)	51 6 ± 13	60	all patients undergoing hepatic angiography and intervention for HAS after liver transplantation	2009–2016	1129	6.9% (79/1129)	71 days	Although endovascular treatment of HAS is generally safe, vessel injury can occur, especially in cases of severe tortuosity or prior retransplantation, increasing complication risk two- to threefold. While endovascular techniques can manage acute injury, these patients remain at high risk for HAT and require close monitoring
**Linda Le et al. 2015** [12]	USA	Retrospective	42 patients: (PTA *n* = 17, stents *n* = 25)	50.9 6 ± 11.3	31	Liver transplant recipients with severe HAS diagnosed via ultrasound	2009–2013	654	6.4% (42 of 654)	82.2 days	HAS after OLT can be treated endovascularly with high technical success and excellent primary assisted patency. Initial use of a stent showed a strong trend toward decreasing the need for reintervention. Avoidance of hepatic artery thrombosis is possible with endovascular treatment and close follow-up
**Hamby et al. 2013** [27]	USA	Retrospective	23 patients (stents *n* = 13, PTA alone *n* = 10)	49.4 ± 6 9	15	Liver transplant recipients with HAS diagnosed via Doppler ultrasound and CTA	2009–2012	318	7.4% (23/318)	92.5 days	Endovascular treatment of HAS can be performed with high technical success, excellent primary-assisted patency, and acceptable morbidity. Initial use of a stent may improve primary patency when compared with PTA. The need for reintervention is common, placing particular importance on aggressive surveillance
**Jarmila et al.2010** [14]	Czech Republic	Retrospective	18 patients, 22EVT (stent *n* = 15, PTA *n* = 6)	42(range, 3–62)	13	Liver transplant recipients with HAS.	1996–2010	800	2.25% (18/800)	82.18 days	Percutaneous angioplasty/stent placement is a safe method for the treatment of hepatic artery stenosis after orthotopic liver transplantation, with a high technical success rate and promising mid-term results
** Sabri et al. 2011 ** [24]	USA	Retrospective	25 patients, (PTA *n* = 21, stent *n* = 4)	51 (range, 17–72).	NA	patients after liver transplantation with HAS or HAT	1997–2009	NA	25	103 days	Endovascular management is effective for HAS but not for HAT
**Maruzzelli et al.2010** [28]	USA	Retrospective	15 patients (PTA *n* = 6, stent *n* = 9)	48 (range 19–68)	10	Liver transplant recipients with HAS	2003–2009	404	3.7% (15/404)	68.13 days	Percutaneous interventional treatment of HAS in LT recipients is safe and effective and decreases the need for surgical revascularization and liver retransplantation
**Bommenaa ET AL. 2022** [22]	USA	Retrospective	63 patients: 44 EVT (PTA alone *n* = 7, stent =37), and 19 No EVT	59 years (IQR 50, 63),	32	adult patients who received Liver transplant with HAS	2013–2018	337	18.96% (63/337)	68 days	EVT was not associated with reduction in HAT progression. HAS has poor graft and overall survival

Abbreviations: *CTA* Computed Tomography Angiography, *EVT* Endovascular Treatment, *HAT* Hepatic Artery Thrombosis, *HAK* Hepatic Artery Kinking, *HAS* Hepatic Artery Stenosis, *IQR* Interquartile Range, *LT* Liver Transplantation, *NA* Not Available, *OLT* Orthotopic Liver Transplantation, *PTA* Percutaneous Transluminal Angioplasty, *RI* Resistive Index, *SD* Standard Deviation

The incidence of HAS varied across studies, ranging from 2.4% in Jarmila et al. [14] to 18.96% in Bommenaa et al. [29]. Patient ages spanned from 42 to 59 years. Geographically, the studies were conducted in the USA (*n* = 6), France (*n* = 2), and the Czech Republic (*n* = 1). Regarding procedural access, femoral access was the preferred approach in most cases. However, in patients with marked caudal angulation of the celiac artery, the brachial artery was used as an alternative approach. A comprehensive summary of study characteristics is provided in Table 1. The NOS assessment showed that all included studies were of good quality (Supplementary File Table 1).

Based on our inclusion criteria, we included studies focusing on adult patients with HAS and excluded those involving pediatric populations. However, Maruzzelli et al. [28] and Jarmila et al. [14] included both adult and pediatric patients. To maintain consistency, we extracted and analyzed data exclusively from the adult patients in these studies.

The technical aspects of endovascular management for HAS are summarized in Table 2, outlining diagnostic methods, treatment strategies, stent types, vascular access routes, and essential procedural equipment including guiding catheters, wires, and balloon catheters.

**Table 2:** Technical characteristics, diagnostic tools, and therapeutic strategies of endovascular management for hepatic artery stenosis in liver transplant recipients

Author (Year)	Diagnostic Tools for HAS	Therapeutic Strategy & Decision Criteria	Stent Type	Indication for Stent Type Selection	Number of Patients Using Femoral or Brachial Access	Guiding Catheter/Sheath	Wire(s) Used	Balloon Catheters (Size/Type
**Bommenaa et al. (2022)** [22]	- DUS: RI < 0.5, PSV > 200 cm/s, tardus parvus - CTA: > 50% luminal narrowing - 82.5% diagnosed by CTA	- Conservative for asymptomatic cases - EVT: • PTA only if tortuous vessels or origin stenosis • Stenting preferred unless anatomy unsuitable	- Covered stents (most common) - Uncovered body stents Cardiac & neurointerventional stents	NA	NA	NA	NA	NA
**Goldsmith et al. (2017)** [26]	- DUS: PSV > 400 cm/s, RI < 0.5, tardus parvus waveform - CTA in normal renal function - Diagnostic angiography if needed	- Started with PTA - Stenting if > 30% residual stenosis - Reintervention after failed PTA always with stenting	- > 95% coronary balloon-expandable - 18% drug-eluting - Covered stents for rupture	NA	Femoral access was used for 77 of 106 (72.6%) angiograms. Brachial access was used when there was marked caudal angulation of the celiac artery.	6 F RDC or RDC-1 (femoral), 5 F multipurpose sheath (brachial)	0.035″ stiff angled Glidewire, 0.014″ guidewire (regular or ChoICE PT)	Coronary angioplasty balloons 2.0–5.0 mm diameter, 12–30 mm length
**Hamby et al. (2013)** [27]	- DUS: RI < 0.5, PSV > 400 cm/s, tardus parvus waveform - CTA: for anatomical details - DSA: final confirmation; < 30% residual stenosis = success	- PTA alone if residual stenosis < 30% - Provisional or primary stenting if residual stenosis > 30%	- Self-expanding - Balloon-expandable - Drug-eluting for recurrent cases	Self-expanding stents: Used when there was significant size mismatch between proximal and distal hepatic artery segments Initially chosen for tortuous or angulated vessels due to better conformability Limited by poor trackability in tortuous anatomy, even with stiff or buddy wires Balloon-expandable coronary stents: Preferred in tortuous anatomy due to superior trackability Use increased with operator experience Drug-eluting stents: selectively used for recurrent stenosis	Femoral access was favored unless there was significant caudal orientation of the celiac artery on the CTA; in these cases, left brachial access was used.	NA	Stiff wires used; buddy wire used for self-expanding stents	PTA routinely used for predilation; PTA alone if residual < 30%
**Jarmila et al. (2010) **[14]	-CDUS: routine noninvasive screening for high flow velocity or low resistance index; monthly follow-up first year, then every 3 months - CTA: > 50% vessel narrowing; pre-PTA assessment of vascular anatomy and stenosis location - MRA: visualize graft and vascular anastomosis	-PTA and stent placement primary treatments -PTA alone preferred in tortuous vessels where stenting difficult - Stenting used if balloon dilatation insufficient - Tortuosity influences choice: PTA alone if high tortuosity; stent if recoil or failure	- Self-expandable (Smart, Wallstents) - Balloon expandable (Express, Palmaz Genesis, AVE coronary, Pro-Kinetic Energy) - No specification on bare, drug-eluting, or covered	NA	Transfemoral approach: 17 procedures Left brachial artery: 4 cases Surgical cut-down: 1 hepatic artery stent (due to extreme arterial tortuosity)	NA	NA	NA
Khati et al. (2022) [25]	- CDUS: PSV > 200 cm/s, SAT > 10 ms, RI < 0.5 - CT/MRI for confirmation - Angiography for final confirmation - > 70% narrowing = HAS	- PBA alone if < 30% residual stenosis - Stenting if > 30% residual stenosis or complications (dissection, rupture) - Primary stenting or post-PBA depending on anatomy/operator	- Balloon-expandable (Palmaz Blue, Cordis) - Rebel (Boston Scientific) - Covered stent for dissection	NA	All patients underwent femoral access, with no cases of brachial access reported.	6 F RDC catheter, 5 F Cobra catheter	0.035″ guidewire initially, then 0.014″ or 0.0016″ guidewire with/without microcatheter	Initial balloons 4–5 mm (1 mm smaller than vessel), larger if needed
**Linda Le et al. (2015)** [12]	- CDUS: RI < 0.5, PSV > 400–450 cm/s, tardus parvus - CTA for confirmation - DSA during intervention	- Historical use of PTA - Shift toward primary stenting due to rapid restenosis with PTA - Recurrent stenosis treated with stenting	- Coronary balloon-expandable (preferred) - Drug-eluting for specific cases - Bare-metal stents	Coronary balloon-expandable stents were generally favored due to: Superior trackability Precision of deployment Suitability for tortuous arteries and focal lesions Drug-eluting coronary stents used in: -Recurrent stenosis -Small-caliber vessels	Access selection depended on the celiac artery’s orientation. A brachial approach was preferred for acutely angled takeoffs, while a perpendicular origin favored femoral access. The femoral approach used in most cases	Multipurpose long sheath or guide	0.014″ wire to cross lesion	Low-profile coronary balloons 2.0–5.0 mm diameter, 15–30 mm length
**Magand et al. (2019)** [11]	- Serial US post-op: days 1, 3, 7, then long-term - US: RI < 0.5, SAT > 80 ms, tardus parvus - CT angiography for confirmation	- PTA for short, concentric HAS or proximal tortuosity - Stenting for long/irregular stenosis, HAK, PTA failure, or dissection - Primary stenting preferred for complex HAS	- Balloon-expandable - No specific type detailed (bare/drug-eluting/covered)	NA	All patients underwent femoral access, with no cases of brachial access reported.	6 F support sheath (Destination or Launcher)	0.014″ guide wire (Galeo, Runthrough)	Coronary monorail balloon 3–6 mm diameter, 20 mm length
**Maruzzelli et al. (2010)** [28]	- DUS daily for 7 days, then interval-based - MDCT for suspicious US - DSA confirms; TPG used	- PTA or stenting based on post-angiographic decision	- Covered balloon-expandable and uncovered stents depending on lesion	NA	All patients underwent femoral access, with no cases of brachial access reported.	4 F or 5 F Cobra 2 or SOS catheters; microcatheter used	0.018″ or 0.014″ stiff wire	High-pressure balloons 2.5–5 mm diameter
**Sabri et al. (2011)** [24]	- DUS within 4 weeks post-op, then annually - DSA, CTA, MRA as needed	- PTA primarily - Stenting for residual stenosis > 30% or flow-limiting dissection	- Bare metal (self-expanding/balloon-expandable) - 1 covered stent for perforation	NA	NA	6-F introducer sheath in CFA; 5-F diagnostic catheter used with hydrophilic guidewire.	Hydrophilic 0.035″ wire to cross stenosis; 0.018″ or 0.014″ wire used for balloon angioplasty.	Balloon diameter sized to 100–120% of normal adjacent HA; specific balloon types not mentioned.

Abbreviations: *DUS* Doppler Ultrasound, *RI* Resistive Index, *PSV* Peak Systolic Velocity, *CTA* Computed Tomography Angiography, *EVT* Endovascular Therapy, *PTA* Percutaneous Transluminal Angioplasty, *CDUS* Color Doppler Ultrasound, *MRA* Magnetic Resonance Angiography, *DSA* Digital Subtraction Angiography, *SAT* Systolic Acceleration Time, *PBA* Percutaneous Balloon Angioplasty, *HAK* Hepatic Artery Kinking, *HAS* Hepatic Artery Stenosis, *HA* Hepatic Artery, *TPG* Transhepatic Pressure Gradient, *US* Ultrasound, *MDCT* Multidetector Computed Tomography, *F* French, *RDC* Renal Double Curve, *CFA* Common Femoral Artery, *SOS* Sidewinder Occlusion Support, *0.035″, 0.018″, 0.014″, 0.0016″* wire diameters in inches

### Anticoagulation methods

Across the reviewed studies, anticoagulation protocols varied slightly depending on the type of intervention and stent used (Table 3). Most studies reported administering intra-procedural weight-based bolus heparin ranging from 50 to 100 units/kg. Some protocols also included additional subcutaneous heparin doses post-procedure.

**Table 3:** Protocols of anticoagulation around the time of percutaneous procedures

Author (Year)	Intra-Procedural Anticoagulation	Post-Procedural Antiplatelet Therapy
**Bommena et al. (2022)** [22]	Not specified	Dual antiplatelet therapy (aspirin + clopidogrel)
**Goldsmith et al. (2017)** [26]	Heparin 100 units/kg (weight-based bolus)	- PTA only: Aspirin 81 mg - Bare metal stents: Aspirin 81 mg + clopidogrel 75 mg for 1 month; aspirin continued long-term - Drug-eluting stents: DAPT (aspirin + clopidogrel) for 6–12 months; aspirin continued long-term
**Hamby et al. (2013)** [27]	Not specified	DAPT (aspirin 81 mg + clopidogrel 75 mg) for 3–6 months; clopidogrel stopped after 6 months if no restenosis
**Jarmila et al. (2010) **[14]	Heparin 5000 units during procedure + 5000 units SC post-procedure (evening and next morning)	Aspirin 100 mg daily for at least 3 months
**Khati et al. (2020)** [25]	Heparin 50 units/kg (weight-based bolus)	- PTA only: Aspirin + clopidogrel for 6 weeks - Stents: Lifelong aspirin + clopidogrel for 1 year
**Linda Le et al. (2015)** [12]	Not specified	DAPT for 3–6 months
**Magand et al. (2019)** [11]	Heparin 50 units/kg (weight-based bolus)	Lifelong aspirin 75 mg post-liver graft+ clopidogrel 75 mg for 1 month if stent was placed
**Maruzzelli et al. (2010)** [28]	Heparin 2000–3000 IU	Aspirin maintained for 1 year
**Sabri et al. (2011)** [24]	Weight-based bolus heparin	Aspirin 81–325 mg/day for life+ clopidogrel 75 mg/day for at least 30 days if stent placed

Abbreviations: *PTA* Percutaneous Transluminal Angioplasty, *DAPT* Dual Antiplatelet Therapy, *ASA* Acetylsalicylic Acid, *SC* Subcutaneous, *EVT* Endovascular Treatment, *IU* International Units

Post-intervention, dual antiplatelet therapy (DAPT) with aspirin (81–100 mg) and clopidogrel (75 mg) was the most common regimen. The duration of DAPT varied based on the type of intervention: patients treated with PTA or bare metal stents typically received DAPT for 1 to 3 months, whereas drug-eluting stents required prolonged DAPT for 6 to 12 months. Long-term monotherapy with aspirin was generally continued in all patients. In liver transplant recipients, lifelong aspirin therapy was used, with the addition of clopidogrel for 1 month in those receiving stents.

### Primary outcomes

#### Primary patency rate

**Fig. 2:**
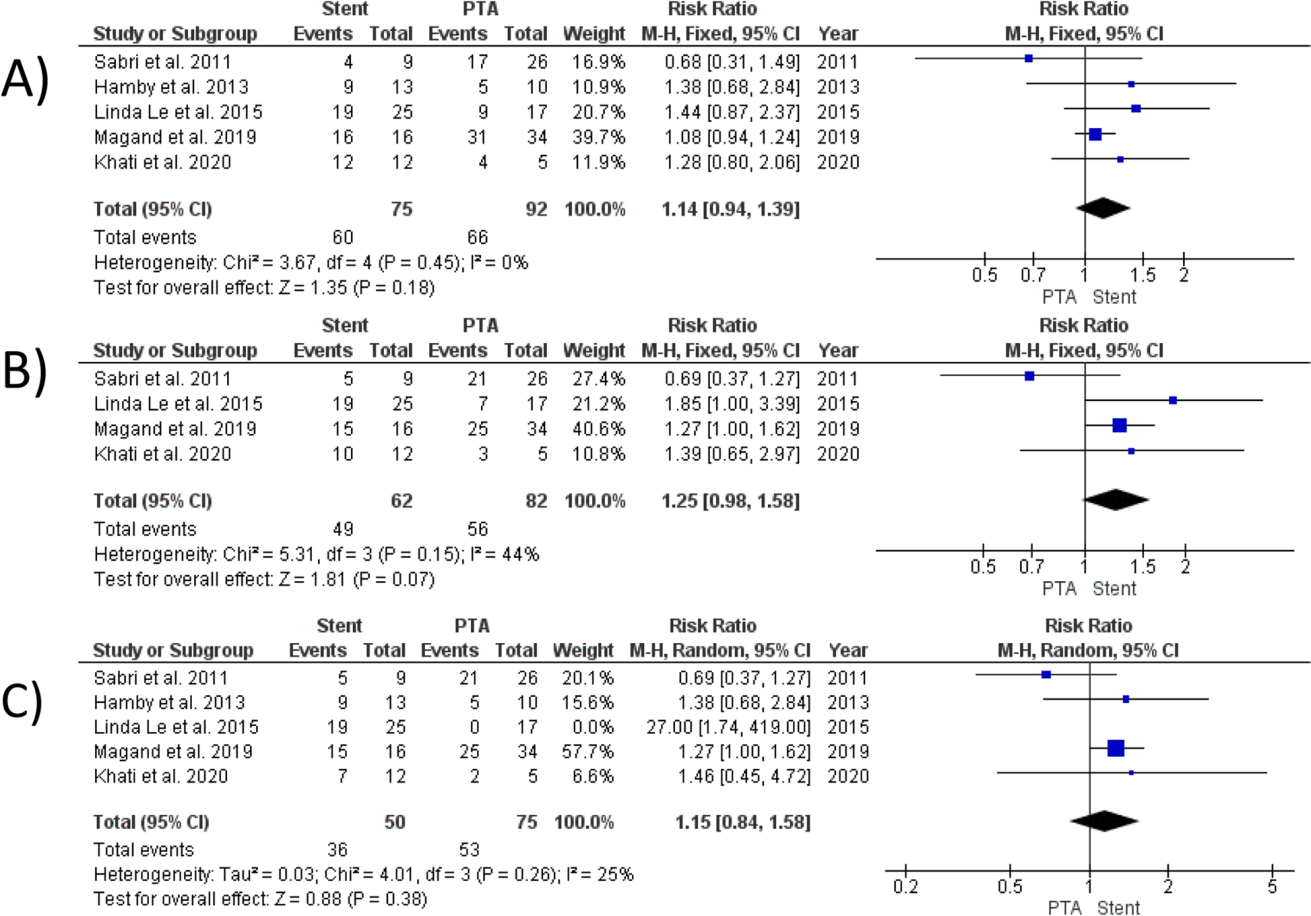
Forest plot comparing Stent versus PTA for primary patency rate at 6 month (**A**),12 months (**B**) and the end of follow up (**C**)

Our analysis found no significant difference between PTA alone and stenting in patency rates at 6 months (RR = 1.14, 95% CI [0.94, 1.39], *P* = 0.18) (Fig. 2A), 12 months (RR = 1.25, 95% CI [0.98, 1.58], *P* = 0.07) (Fig. 2B), and at the end of follow-up (RR = 1.15, 95% CI [0.84, 1.58], *P* = 0.38) (Fig. 2C). The analysis demonstrated homogeneity at 6 months (I^2^ = 0%, *P* = 0.45) and 12 months (I^2^ = 44%, *P* = 0.15). However, significant heterogeneity was observed in the analysis of the end of follow-up (I^2^ = 71%, *P* = 0.008). This heterogeneity was resolved by excluding the study by Linda Le et al. [12] (I^2^ = 25%, *P* = 0.26).

### Secondary outcomes

#### Need for reintervention

**Fig. 3:**
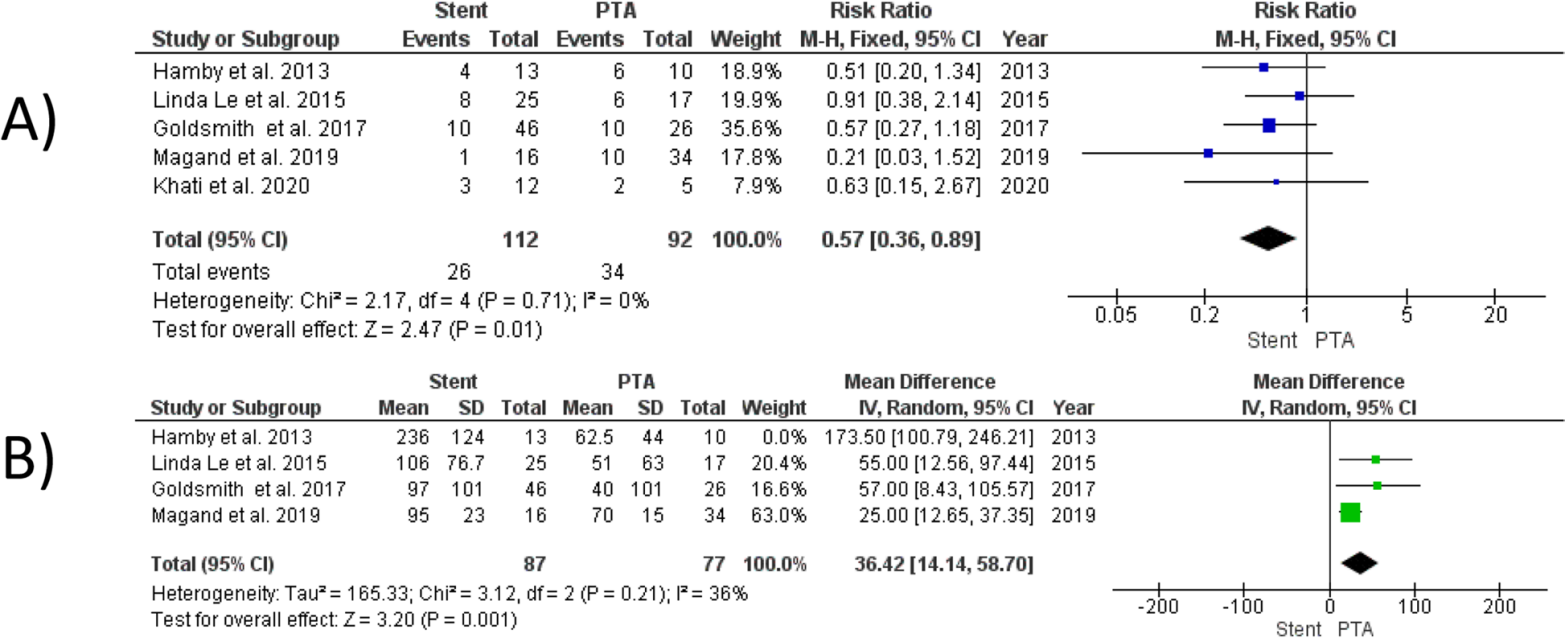
Forest plot comparing Stent versus PTA for need for reintervention (**A**), and time to recurrent HAS (**B**)

Our analysis revealed that stenting was significantly associated with lower incidence of reintervention compared to PTA alone (RR = 0.57, 95% CI [0.36, 0.89], *P* = 0.01). The analysis demonstrated homogeneity (I^2^ = 0%, *P* = 0.71) (Fig. 3A).

#### Time to recurrent HAS

Our analysis showed that stenting was significantly associated with a longer time to recurrent HAS compared to PTA alone (MD = 36.42 days, 95% CI [14.14, 58.70], *P* = 0.001). The analysis exhibited substantial heterogeneity (I^2^ = 83%, *P* = 0.0005). However, excluding the study by Hamby et al. [27] resolved the heterogeneity, resulting in a more homogeneous analysis (I^2^ = 36%, *P* = 0.21) (Fig. 3B).

#### Technical success rate

**Fig. 4:**
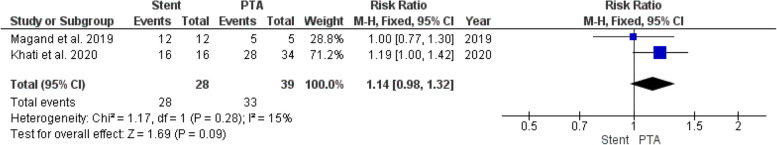
Forest plot comparing Stent versus PTA for technical success rates

Our analysis found no significant difference in technical success rates between stenting and PTA alone (RR = 1.14, 95% CI [0.98, 1.32], *P* = 0.09) (Fig. 4). The analysis demonstrated homogeneity (I^2^ = 15%, *P* = 0.28)**.**

#### Major complications

**Fig. 5:**
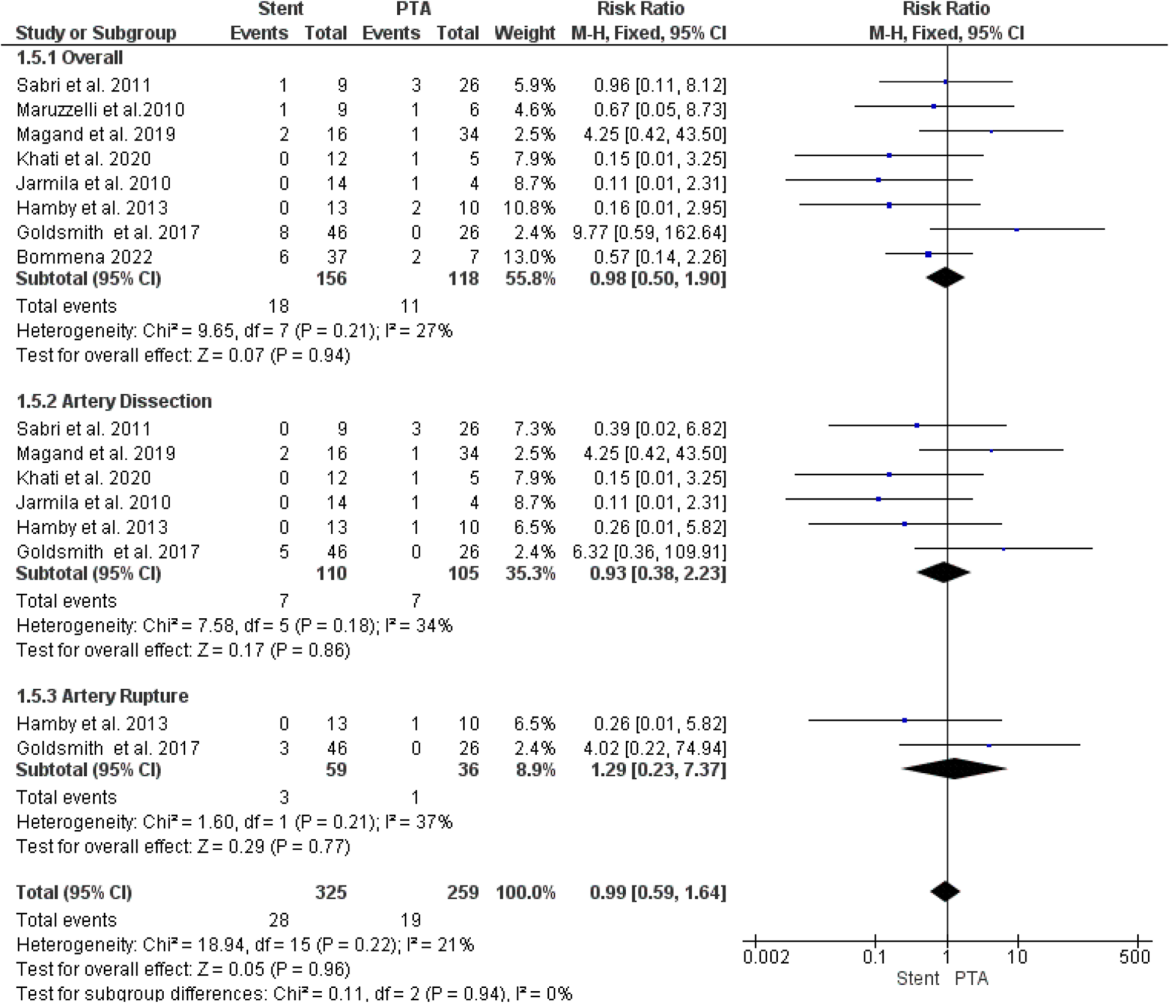
Forest plot comparing Stent versus PTA for major complications and its subgroup analysis artery dissection and artery rupture

Our analysis found no significant difference in major complication rates between stenting and PTA alone (RR = 0.98, 95% CI [0.50, 1.90], *P* = 0.94). The analysis demonstrated homogeneity (I^2^ = 27%, P = 0.21) (Fig. 5).

Our subgroup analysis, stratified by major complication type, showed no significant differences between stenting and PTA alone in the risk of artery dissection (RR = 0.93, 95% CI [0.38, 2.23], *P* = 0.86) or rupture (RR = 0.99, 95% CI [0.59, 1.64], *P* = 0.77) (Fig. 5). Both analyses demonstrated homogeneity, with I^2^ = 34% (*P* = 0.18) for dissection and I^2^ = 37% (P = 0.21) for rupture.

#### Incidence of hepatic artery thrombosis

**Fig. 6:**
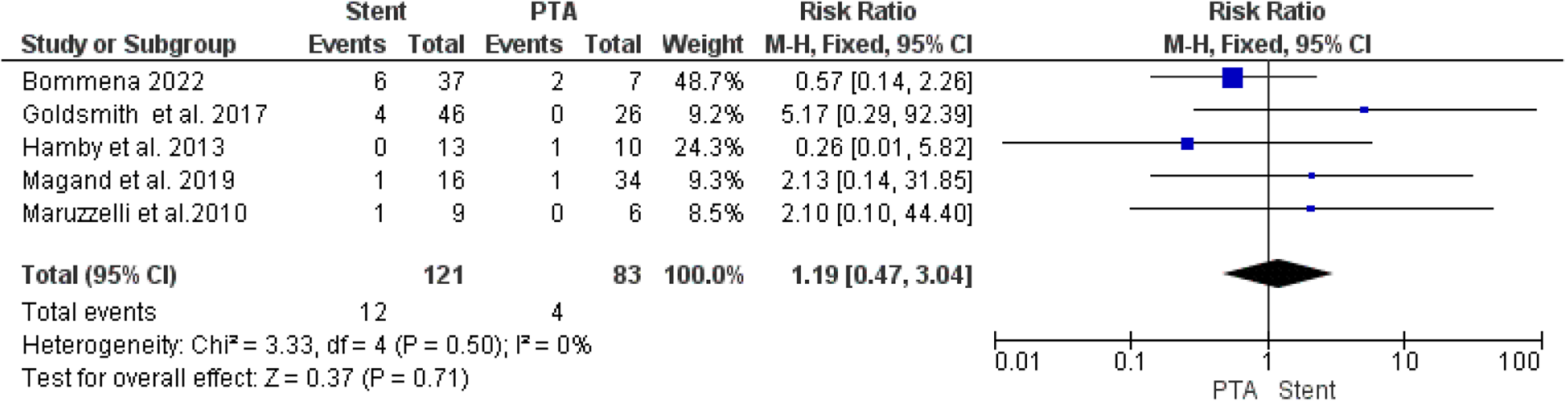
Forest plot comparing Stent versus PTA for incidence of Hepatic artery thrombosis

Our analysis found no significant difference in HAT rates between stenting and PTA alone (RR = 1.19, 95% CI [0.47, 3.04], *P* = 0.71) (Fig. 6). The analysis demonstrated homogeneity (I^2^ = 0%, *P* = 0.50).

## Discussion

This meta-analysis of nine studies, including 325 patients with HAS after liver transplantation, revealed no significant differences between stenting and PTA in primary patency rate at 6 months, 12 months, or the end of follow-up. Additionally, there were no significant differences in major complications, artery dissection, artery rupture, incidence of HAT, or technical success rates. However, stenting was associated with a significantly lower reintervention rate and a longer time to recurrent HAS compared to PTA.

HAS occurs at the anastomotic site in approximately 70% of cases, typically within three months post-transplant, causing arterial stagnation, thrombosis, hepatic necrosis, and bile duct ischemia, ultimately compromising graft function [2, 30–32]. Surgical management of HAS carries a high risk of complications, especially in liver transplant recipients with critical conditions. While Abbasoglu favored surgery for long-segment stenosis [33], Saad et al. argued that interventional treatments provide similar outcomes with better safety, noting that surgery can lead to hepatic artery thrombosis in up to 26% of cases [8].

This meta-analysis found no significant difference between stenting and PTA in maintaining hepatic artery patency at 6 months, 12 months, and the end of follow-up, suggesting comparable effectiveness in preventing further intervention. Our findings align with those of Khati et al. [25], Magand et al. [11], Hamby et al. [27], Sabri et al. [24], Linda Le et al. [12], and the meta-analysis by Rostambeigi et al. [18].

However, this challenges the notion that stenting is superior to PTA, as Magand et al. [11] and Hamby et al. [27] reported higher rates of recurrent HAS after PTA. This may explain why some clinicians prefer stenting as a first-line treatment to improve long-term arterial patency. One possible explanation for the comparable outcomes in our analysis is the small sample size in the included studies, which could lead to a type II error, masking a true difference between the two interventions. Additionally, the heterogeneity of HAS, including variations in location, length, and morphology, may influence treatment outcomes, as the optimal approach often depends on these anatomical factors.

Despite this, our analysis demonstrated a significant reduction in reintervention rates with stenting compared to PTA, contradicting previous studies [11, 12, 25–27] and meta-analysis by Rostambeigi et al. [18] that found no significant difference between the two interventions. Additionally, stenting was associated with a significantly longer time to recurrent HAS, suggesting potential long-term benefits.

This advantage may be attributed to the mechanical support provided by stents, which act as a structural scaffold within the hepatic artery at the site of stenosis. By maintaining the widened lumen achieved during initial angioplasty, stents help counteract the vessel’s natural elastic recoil, which can lead to early restenosis after PTA alone. The sustained luminal patency provided by stents may reduce the likelihood of early restenosis and, consequently, the need for reintervention [16, 29].

Furthermore, some treatment protocols [25, 26] recommend stenting in cases where residual stenosis exceeds 30% after PTA or when complications such as dissection or rupture occur. This selective use of stents to manage suboptimal PTA outcomes or procedural complications may contribute to improved immediate and long-term results, ultimately influencing reintervention rates.

Moreover, Magand et al. [11] highlighted the impact of HAS morphology on recurrence rates, showing that PTA alone was particularly prone to restenosis in complex cases, such as HAS on a hepatic artery kink (HAK) or long, squeezed HAS. Notably, restenosis rates were 30% for HAS on HAK, 50% for squeezed HAS on HAK, and 33.3% for long, squeezed HAS, whereas no restenosis occurred after stenting in these cases [11]. These findings, consistent with Saad et al., suggest that stenting may be a superior approach for HAS associated with HAK, offering better technical success and lower complication rates [7, 12].

Initially, self-expanding stents were selected for their ability to better conform to vessel curves and potentially minimize distal kinking [12, 27]. However, their poor trackability in tortuous hepatic arteries, even with techniques such as buddy wires or stiff wires, limited their practical use [12, 27]. Coronary balloon-expandable stents are now more commonly used in these situations due to their enhanced navigability and deployment precision [12].

Multiple studies have emphasized that vessel tortuosity not only complicates the technical performance of PTA but also increases the risk of complications and restenosis [11, 26, 27]. Therefore, in anatomically complex HAS, especially those involving HAK or long, tortuous segments, stenting should be considered the preferred first-line treatment. PTA alone may be reserved for simpler lesions, such as short, concentric stenoses without vessel tortuosity or in cases where stent delivery is technically unfeasible due to extreme angulation.

Another key consideration when selecting a treatment approach for HAS EVT is the anatomy of the celiac trunk and hepatic artery [11]. Following OLT, the hepatic artery often exhibits significant tortuosity, which can make catheterization and advancing a stent to the site of stenosis challenging [11]. While stent placement appears to offer better long-term patency for HAS occurring on a HAK, navigating a stent across a HAK can be technically demanding and, in some cases, unfeasible. This procedure requires a learning curve and carries the risk of complications such as vascular dissection or HAT [11].

Bommena et al. [29] investigated factors contributing to the development of HAS and decisions regarding its management. Their findings indicated that female gender, pre-transplant portal vein thrombosis, and diabetes were significantly associated with the need for EVT [29]. Estrogen exposure and underlying hypercoagulable states may impair vascular patency, potentially contributing to the pathogenesis of HAS in these populations [34, 35]. Moreover, small vessel arteriolosclerosis, commonly observed in diabetics and women, could further predispose patients to vascular compromise [36]. While these associations suggest a biological basis for sex and comorbidity-related variations in HAS, their impact on treatment outcomes across different modalities such as PTA, stenting, or anticoagulant therapy remains uncertain [29]. Further research is needed to clarify whether estrogen-related or hypercoagulable conditions influence therapeutic decisions or predict the success of specific interventions.

Regarding major complication, our analysis found comparable rates between stenting and PTA, consistent with previous studies [11, 12, 25–27] and meta-analysis by Rostambeigi et al. [18]. Additionally, our subgroup analysis by complication type showed no significant differences in artery rupture or dissection rates between the two interventions. One possible explanation is that severe vessel tortuosity and the presence of a second liver transplant may contribute to complications associated with endovascular treatment for HAS [26]. Saad et al. reported that patients with arterial kinks or tortuosity experienced lower technical success and higher complication rates with PTA, particularly in cases involving tandem or distal stenosis. Their study found that patients with vessel tortuosity had a technical success rate of only 14% and a complication rate of 29%, compared to 94% and 10%, respectively, in those without such anatomical challenges [12]. The increased complication risk in retransplant patients remains unclear but may be attributed to technical difficulties in arterial anastomosis, compromised vessel quality due to chronic inflammation, or the long-term effects of immunosuppression and steroid therapy [26].

However, it is important to note that Goldsmith et al. reported a significantly higher risk of HAT in patients who experienced complications during the initial endovascular treatment of HAS [26]. Despite successfully managing 75% of these complications, the affected patients had a 50% risk of developing HAT, compared to just 1.4% in those who underwent uncomplicated treatment [26]. This highlights the critical impact of procedural complications on long-term outcomes, emphasizing the need for meticulous technique and careful patient selection to minimize risks during endovascular treatment of HAS.

Lastly, our analysis found comparable HAT rates between stenting and PTA. This may be due to factors such as prolonged cold ischemia time, increased bleeding during arterial reconstruction, poor primary graft function, and complex arterial reconstructions, all of which can impair intrahepatic blood flow and contribute to HAT development [12, 37]. While prophylactic low-dose aspirin has been shown to reduce late HAT in post-transplant patients [11, 38], Wolf et al. reported that it did not prevent HAT in those with HAS [39]. Similarly, Bommena et al. found no significant reduction in HAT incidence despite the use of antiplatelet and anticoagulant therapy in HAS patients undergoing EVT [29]. These findings suggest that HAS may progress to HAT despite successful EVT and antiplatelet therapy, indicating a more complex underlying pathophysiology.

### Implications and future directions

To optimize treatment strategies for HAS, future research should aim to identify patient and lesion characteristics that predict favorable outcomes with specific techniques. Prospective studies comparing different stent types and HAS morphologies, while evaluating long-term clinical endpoints such as graft function, patient survival, and quality of life, are essential to inform and refine clinical decision-making. Additionally, reporting outcomes stratified by stenosis morphology would support the development of tailored treatment algorithms. Establishing standardized treatment protocols and fostering multidisciplinary collaboration between transplant surgeons and interventional radiologists may further improve outcomes by promoting a personalized and evidence-based approach. Furthermore, dedicated investigations into the clinical efficacy and long-term durability of drug-eluting stents in HAS management are warranted to determine their optimal role.

### Limitations

Our study has several limitations. First, we exclusively included English-language studies, which may introduce publication bias. Additionally, all included studies were retrospective, making them susceptible to inherent biases such as selection bias. Moreover, variations in resource availability and differences in medical expertise across institutions may have influenced treatment decisions, further constraining the broader applicability of our results. Another limitation is that we were unable to assess important clinical outcomes, such as quality of life, liver function post-treatment, re-transplantation rates, mortality, and patient survival, as these data were not reported in the included studies. Furthermore, our ability to conduct subgroup analyses was restricted, preventing us from comparing different types of stents (bare-metal vs. covered, balloon-expandable vs. self-expandable), lesion characteristics (short vs. long segment HAS, tortuous vs. non-tortuous vs. tandem lesions), and timing of presentation (early vs. late HAS). Lastly, none of the included studies provided detailed morphological descriptions of the stenosis, such as hepatic artery kinking or external compression, which restricted our ability to evaluate treatment efficacy based on anatomical variations.

## Conclusion

Our findings indicate that both techniques achieve comparable primary patency rates and safety outcomes, making them viable treatment options. However, stenting was associated with a significantly lower reintervention rate and a longer time to recurrent HAS, suggesting potential advantages in long-term durability.

## Supplementary Information

Supplementary Material 1.

## Data Availability

All data generated or analyzed during this study are included in this published article or in the data repositories listed in References.
