# Supplementary material for: Percutaneous transluminal angioplasty vs. stenting for hepatic artery stenosis after liver transplantation in adults: a systematic review and meta-analysis
Source: CVIR Endovasc. 2025 Oct 7;8:78. doi: 10.1186/s42155-025-00581-8 (PMC12501083; doi:10.1186/s42155-025-00581-8)
Supplement: Supplementary file 1 — Supplementary Material 1. [file 42155_2025_581_MOESM1_ESM.docx]

**Percutaneous Transluminal Angioplasty vs. Stenting for Hepatic Artery Stenosis After Liver Transplantation in Adults: A Systematic Review and Meta-Analysis**

1. **Search terms and results in different databases**

| Database | Search strategy | Number of Studies | Search date |
| --- | --- | --- | --- |
| PubMed | (Stent* OR Angioplast* OR Endoluminal Repair* OR Transluminal Angioplast* OR Percutaneous Transluminal Angioplast* OR endovascular treatment* OR vascular stenting OR arterial stenting OR balloon angioplast*) AND (Liver Transplantation* OR Hepatic Transplantation* OR Liver Transplant* OR Liver Grafting OR orthotopic liver transplantation* OR post-transplant complication*)  AND (Hepatic Artery OR Hepatic Artery Stenosis OR Hepatic Artery Narrowing OR hepatic artery thrombosis OR hepatic artery occlusion) | 427 | January 28, 2025 |
| Scopus | TITLE-ABS-KEY (( stent* OR angioplast* OR "Endoluminal Repair" OR "Transluminal Angioplasty" OR "Percutaneous Transluminal Angioplasty" OR "endovascular treatment" OR "vascular stenting" OR "arterial stenting" OR "balloon angioplasty" ) AND ( "Liver Transplantation" OR "Hepatic Transplantation" OR "Liver Transplant" OR "Liver Grafting" OR "orthotopic liver transplantation" OR "post-transplant complications" ) AND ( "Hepatic Artery" OR "Hepatic Artery Stenosis" OR "Hepatic Artery Narrowing" OR "hepatic artery thrombosis" OR "hepatic artery occlusion" ) ) | 522 |  |
| WOS | ALL ((Stent* OR Angioplast* OR Endoluminal Repair* OR Transluminal Angioplast* OR Percutaneous Transluminal Angioplast* OR endovascular treatment* OR vascular stenting OR arterial stenting OR balloon angioplast*) AND  (Liver Transplantation* OR Hepatic Transplantation* OR Liver Transplant* OR Liver Grafting OR orthotopic liver transplantation* OR post-transplant complication*)  AND (Hepatic Artery OR Hepatic Artery Stenosis OR Hepatic Artery Narrowing OR hepatic artery thrombosis OR hepatic artery occlusion)) | 532 |  |
| Cochrane CENTRAL | ((Stent* OR Angioplast* OR Endoluminal Repair* OR Transluminal Angioplast* OR Percutaneous Transluminal Angioplast* OR endovascular treatment* OR vascular stenting OR arterial stenting OR balloon angioplast*) AND (Liver Transplantation* OR Hepatic Transplantation* OR Liver Transplant* OR Liver Grafting OR orthotopic liver transplantation* OR post-transplant complication*) AND (Hepatic Artery OR Hepatic Artery Stenosis OR Hepatic Artery Narrowing OR hepatic artery thrombosis OR hepatic artery occlusion)) in Title Abstract Keyword | 8 |  |

***Supplementary Table 1. Methodological quality assessment of the included 9 studies, based on the NOS for assessing the quality of observational studies.***

1. Cohort studies (n=9)

| Study | Selection | | | | Comparability | Outcome | | | Total Score |
| --- | --- | --- | --- | --- | --- | --- | --- | --- | --- |
|  | Representativeness of the exposed cohort | Selection of the non-exposed cohort | Ascertainment of exposure^5^ | Outcome was not present at start of study^6^ | Control for 2 important factors^2,3^ | Assessment of outcome | Follow-up long enough | Adequacy of follow-up of cohort^7^ |  |
| Khati et al. 2020 | ★ | ★ | ★ |  |  | ★ | ★ | ★ | 7 |
| Magand et al. 2019 | ★ | ★ | ★ |  | ★ | ★ | ★ | ★ | 8 |
| Goldsmith et al. 2017 | ★ | ★ | ★ |  |  | ★ | ★ | ★ | 6 |
| Linda Le et al. 2015 | ★ | ★ | ★ |  |  | ★ | ★ | ★ | 7 |
| Hamby et al. 2013 | ★ | ★ | ★ |  |  | ★ | ★ | ★ | 6 |
| Jarmila et al. | ★ | ★ | ★ |  | ★ | ★ | ★ | ★ | 8 |
| Sabri et al. 2011 | ★ | ★ | ★ |  | ★ | ★ | ★ | ★ | 8 |
| Maruzzelli et al.2010 | ★ | ★ | ★ |  | ★ | ★ | ★ | ★ | 7 |
| Bommenaa et al. 2022 | ★ | ★ | ★ |  | ★ | ★ | ★ | ★ | 7 |

^1^ If the cases data was obtained from records with mentioning the process to extract information, or reference to primary record, a point was assigned.
^2^ If adjusted for age, a point was assigned.
^3^ If adjusted for drugs (e.g. anti-hypertensives, anti-diabetics etc) or any other additional factors, a point was assigned.

^4^ If information were obtained through national registries or hospital records, a point was assigned.
^4^ If information were obtained from patients and the difference in non-response rate between groups was 20% or less a point was assigned.
^5^ If the exposure data was obtained from prescription database or medical record, a point was assigned.
^6^ If the study design is prospective study, a point was assigned.
^7^ If the completeness of follow-up was 80% or more, a point was assigned.
